# MicroRNA-204 inhibits proliferation, migration, invasion and epithelial-mesenchymal transition in osteosarcoma cells via targeting Sirtuin 1

**DOI:** 10.3892/or.2021.8098

**Published:** 2021-05-31

**Authors:** Ying Shi, Jianjun Huang, Jun Zhou, Ying Liu, Xiaodan Fu, Yimin Li, Gang Yin, Jifang Wen

Oncol Rep 34: 399-406, 2015; DOI: 10.3892/or.2015.3986

Following the publication of this paper, it was drawn to the Editors attention by a concerned reader that certain of the cell Transwell assay data in the article (featured in Figs. 3B and 6B) were strikingly similar to data that appearing in different form in another article by different authors at different research institutions, which had already been published elsewhere at the time of the present articles submission.

Owing to the fact that the contentious data in the above article had already appeared in different form in another article prior to its submission to *Oncology Reports*, the Editor has decided that this paper should be retracted from the Journal. The authors did not reply to indicate whether or not they agreed with the retraction of the paper. The Editor apologizes to the readership for any inconvenience caused.

